# Retrospective analysis of the incidence of neurodegenerative disorders in idiopathic rapid eye movement sleep behavior disorder: a preliminary study in Japanese patients

**DOI:** 10.20407/fmj.2019-011

**Published:** 2020-02-11

**Authors:** Reiko Kumagai, Tsuyoshi Kitajima, Marina Hirose, Nakao Iwata

**Affiliations:** Department of Psychiatry, Fujita Health University, School of Medicine, Toyoake, Aichi, Japan

**Keywords:** Rapid eye movement sleep behavior disorder, Neurodegenerative disorders, Incidence, Kaplan–Meier analysis, Japanese

## Abstract

**Objectives:**

Idiopathic rapid eye movement (REM) sleep behavior disorder (iRBD) is characterized by abnormal and potentially violent behaviors during REM sleep, typically observed in older adult subjects. Previous reports have described a high risk for neurodegeneration in patients with iRBD; however, to date, no published study has analyzed an adequate number of Japanese patients. We retrospectively analyzed the incidence of neurodegenerative disorders among patients diagnosed with iRBD in our department.

**Methods:**

The data were retrospectively collected from patients’ medical records. The patients included in the study were diagnosed with iRBD using polysomnography in our department, from May 1, 2005 to November 30, 2018, with a follow-up of ≥6 months. Using the Kaplan–Meier (KM) method, we estimated the incidence of later diagnoses of neurodegenerative disorders among this cohort of patients with iRBD.

**Results:**

Among 57 consecutive patients diagnosed with iRBD, 14 (24.6%) were later diagnosed with neurodegenerative disorders. Using the KM method, we estimated that the incidence was as high as 18.5% and 68.1% at 5 and 10 years, respectively. Of the 14 patients who developed neurodegenerative disorders, 12 (85.7%) had α-synucleinopathies (Parkinson’s disease in eight patients, Lewy body dementia in three, Alzheimer’s-type dementia in two, and multiple system atrophy in one).

**Conclusions:**

The results of this study suggest the high likelihood that iRBD may subsequently progress to neurodegenerative disorders in Japanese patients, a finding similar to those previously reported by studies performed overseas. Further studies using standardized prospective evaluation methods must be performed in Japan.

## Introduction

Rapid eye movement (REM) sleep behavior disorder (RBD) is a parasomnia that is characterized by repeated complex motor behaviors and vocalizations during REM sleep. This disorder is commonly associated with dream mentation during REM sleep and may cause injury or disruptions during sleep.^1^ Although muscle tone is usually lost during REM sleep, REM sleep without atonia (RWA) is recorded on polysomnography (PSG) in patients with RBD. RBD is more commonly observed in men ≥50 years of age. Underlying neurological disorders are often observed in RBD patients, including α-synucleinopathies, such as Parkinson’s disease, Lewy body dementia, and multiple system atrophy; these conditions are considered to be predisposing factors.^2^ However, even in patients with idiopathic RBD (iRBD), without such underlying disorders, neurodegenerative disorders may develop in subsequent years after the onset of iRBD.^2^ In a meta-analysis performed by Galbiati et al., the incidence of neurodegenerative disorders was extremely high (33.5% and 82.4% at 5 and 10.5 years after iRBD diagnosis, respectively).^3^ However, to the best of our knowledge, almost no reports exist regarding the risk of neurodegeneration after iRBD diagnosis in Japanese patients. We aimed to evaluate whether the incidence of neurodegenerative disorders differs on the basis of ethnicity. Identifying what proportion of patients diagnosed with iRBD are likely to subsequently develop neurodegenerative disorders, such as Parkinson’s disease and Lewy body dementia, may contribute to changing how doctors explain the risks of iRBD to patients and how doctors approach follow-up for these patients after diagnosis.

We retrospectively evaluated the subsequent emergence of neurodegenerative disorders in patients with iRBD who were diagnosed and treated at our institution.

## Methods

### Study outline

The study included consecutive patients who visited the Department of Psychiatry at Fujita Health University Hospital, from May 1, 2005 to November 30, 2018, and who were diagnosed with iRBD based on their clinical symptoms and PSG results. The data were collected by retrospectively reviewing the patients’ medical records. The study was performed with the approval of the ethics committee of Fujita Health University. Because this was a retrospective and observational study, the requirement for formal consent from individual subjects was waived.

### Subjects

In this study, the inclusion of patients with iRBD was determined by reviewing the patient medical records in our department. The diagnosis of iRBD was confirmed as described in the following section. All patients who were followed-up for <6 months after the iRBD diagnosis were excluded. Although the presence of comorbid psychiatric diseases was allowed, patients who were suspected to have drug-induced RBD, while on psychotropic medications, were excluded.

### Diagnosis

PSG was performed during routine clinical practice, based on the attending physician’s discretion, according to the method recommended by the American Academy of Sleep Medicine (AASM).^4^ The presence of RWA, based on submental electromyography during the PSG, was manually judged by trained sleep technicians (after the introduction of the 2007 AASM manual for the scoring,^4^ judgments were made according to it), along with the manual scoring of the PSG findings during routine clinical procedures. The diagnosis of RBD was initially made during screening, based on the International Classification of Sleep Disorders (ICSD), at the time of diagnosis. Although the first edition of ICSD did not necessarily require the presence of RWA for RBD diagnosis,^5^ we retrospectively confirmed that all patients presented with RWA according to the PSG reports, and that the diagnostic criteria for RBD presented in the third edition of ICSD (ICSD-3),^1^ the most recent version, were met by all patients included in our study.

To validate the diagnosis of RBD as idiopathic, we made best use of all the information retrieved from the patients’ medical records at the time of the initial diagnosis. We confirmed that there were no descriptions in the records that referred to neurologic symptoms suggesting Parkinsonism, such as muscular rigidity of the extremities, involuntary movements (resting tremor and silent or immobile movement), postural retention reflex disturbances, or obvious cognitive decline. If the Hasegawa Dementia Scale-Revised (HDS-R) or the Mini-Mental State Examination (MMSE) were performed, the cutoff values of HDS-R≤20^6^ and MMSE≤23^7^ were confirmed not to be fulfilled before classifying the diagnosis as idiopathic.

The onset and diagnosis of neurodegenerative disorders employed in this study were determined based on the judgments made by physicians, who were different from the attending physicians who diagnosed iRBD, in the specialized department of neurology or elderly care at our hospital or other institutions, following the natural clinical course. Diagnostic methods, such as the adapted criteria and image testing, were employed at the discretion of each physician in charge at the specialized department.

The comorbidity of other sleep disorders or psychiatric disorders was determined by the attending physicians.

### Analysis

The proportion of patients who were later diagnosed with neurodegenerative disorders was identified. In addition, the incidence of neurodegenerative disorders was estimated using the Kaplan–Meier (KM) method, based on the time elapsed since the diagnosis of iRBD. In this analysis, the date of the neurodegenerative disorder diagnosis was used as the date of “event occurrence” (in cases where patients were diagnosed at other institutions, the date that the patient notified his/her attending physician in our department of the diagnosis was used), and the last visit date for discontinued or ongoing patients without the occurrence of neurodegenerative disorders was used as the date of “censoring.” For each patient, survival data were obtained, using the dates of RBD diagnoses by PSG as the starting point of the analysis. As explorative analyses, differences in survival curves were assessed based on the presence of comorbid depression or sleep apnea. Statistical analyses were performed using JMP 13.0 (SAS Institute Japan, Tokyo, Japan). A *P* value<0.05 was considered to be statistically significant.

## Results

Fifty-seven Japanese patients with iRBD, for whom the presence of RWA on PSG was confirmed, were included in this analysis. None of the patients had apparent neurological symptoms, including Parkinsonism or cognitive decline (as measured by significant decreases in HDS-R or MMSE scores), and no patient was excluded for apparent neurological symptoms. Of the 57 patients, 47 (82.5%) were men and 10 (17.5%) were women. The patients were aged from 43 to 80 (mean: 65.6±7.5 [SD]) years at the time when PSG was performed. The mean age of iRBD onset in this cohort was 61.0±10.0 years; however, we were unable to determine the age of iRBD onset for three patients. Twenty-three patients had comorbid sleep disorders (22 had sleep apnea, one had a disorder of arousal [parasomnia from non-rapid eye movement (NREM) sleep], and one had restless leg syndrome; one patient had two comorbidities [sleep apnea and disorder of arousal]). The PSG results revealed 14 patients with significant periodic limb movements; however, an independent diagnosis of periodic limb movement disorder could not be made because of ICSD-3 in the presence of RBD.^1^ One patient was diagnosed with both RBD and disorder of arousal, in particular, parasomnia overlap disorder, due to the observation of both RWA and strange movements (thrashing the legs) during NREM sleep during the PSG. Six (10.5%) of 57 patients had comorbid mental disorders [major depressive disorder (n=5) and panic disorder (n=1)], whereas the remaining patients (n=51) (89.5%) did not present any mental disorders. The six patients with comorbid mental disorders did not take psychotropic agents, such as antidepressants, which could induce secondary RBD, at the time when PSG was conducted. The mean follow-up period from iRBD diagnosis ranged from 0.5 to 11.4 (mean: 4.0±2.8) years. For patients who were diagnosed with neurodegenerative disorders in other departments, after completing treatment in our department, the time of the neurodegenerative disorder diagnosis in the other department was used as the last observation. A summary of the patients is shown in Table 1.

**Figure 1:**
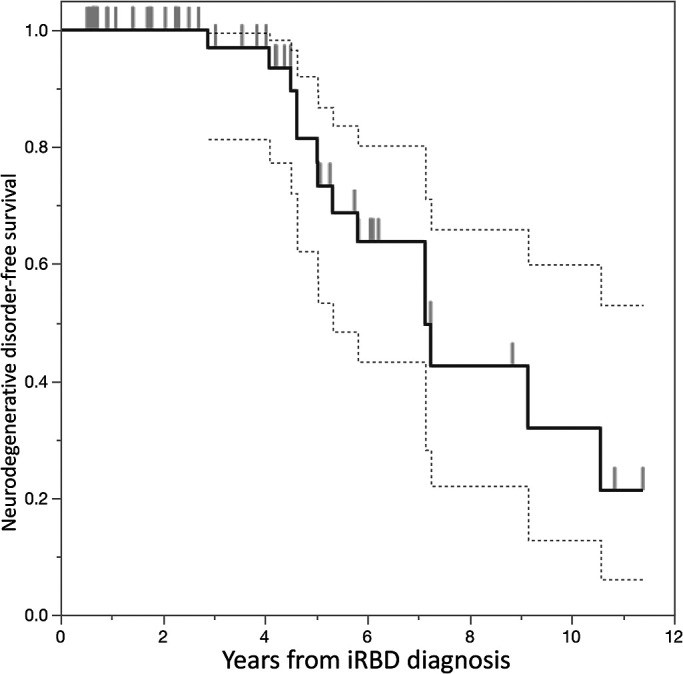
Kaplan–Meier curve of neurodegenerative disorder-free survival of patients with idiopathic rapid eye movement sleep behavior disorder (iRBD). Dotted lines indicate 95% confidence interval.

Neurodegenerative disorders developed in 14 patients, 10 of whom were men and 4 of whom were women, accounting for 24.6% of the total assessed population. The prevalence of neurodegenerative disorders was as follows: Parkinson’s disease in eight patients, Lewy body dementia in three patients, Alzheimer’s-type dementia in two patients, and multiple system atrophy in one patient. The duration between iRBD and neurodegenerative disease diagnoses ranged from 2.9 to 10.6 (mean: 5.9±2.0) years. Using the KM method, a survival curve depicted the relationship between the time since iRBD diagnosis and a predicted survival without the diagnosis of neurodegenerative disorders (Figure 1). Five years from the time of iRBD diagnosis, 18.5% of patients were likely to be diagnosed with neurodegenerative disorders, whereas 10 years from the time of iRBD diagnosis, the percentage of patients likely to be diagnosed with neurodegenerative disorders increased to 68.1%. The differences in the survival curves were not significant when comparing the presence and absence of comorbid depression (*P*=0.9118) or sleep apnea (*P*=0.7521).

## Discussion

Of 57 patients diagnosed with iRBD in the present study, 14 were later diagnosed with neurodegenerative disorders, accounting for 24.6% of the total population. However, using the KM method, 18.5% and 68.1% of patients were likely to be diagnosed with neurodegenerative disorders 5 and 10 years following iRBD diagnosis, respectively. Although a relatively high number of studies have been conducted overseas, only a small number of studies have been performed in Japan. In a systematic review published by Galbiati et al. in 2019, only one study from Japan was included,^3^ in which Sakurai et al. reported that that none of the nine patients, who were followed up for an average of 1.9 years, showed neurological deficits.^8^ Although our study was preliminary and retrospective, we initially assessed a relatively large number of patients who were followed up over a long period of time in Japan, and we succeeded in replicating the possibility that Japanese patients with iRBD might also have a very high risk of developing neurodegenerative disorders within 5–10 years, similar to the risk reported for patients in other countries.

Fourteen patients were later diagnosed with the following neurodegenerative disorders: eight patients with Parkinson’s disease, three patients with Lewy body dementia, two patients with Alzheimer’s-type dementia, and one patient with multiple system atrophy; therefore, 85.7% (12/14) of these patients were diagnosed with an α-synucleinopathy. The dominance of α-synucleinopathies is consistent with the findings from previous reports^3^ and appears to confirm the close relationship between RBD and α-synucleinopathies.

In this study, the incidence of neurodegenerative disorders in participants with iRBD was high; however, we could not compare this incidence with that for participants without iRBD because a control group was not included. Surprisingly, many previous studies have had the same limitation.^3^ Among the general population, Hirsh et al. have reported that, for subjects ≥40 years of age, the incidence of Parkinson’s disease is 0.61 per 1,000 person-years among male subjects and 0.38 per 1,000 person-years among female subjects, based on a meta-analysis.^9^ In a systematic review, Hogan et al. reported that the incidence of Lewy body dementia ranged from 0.5 to 1.6 per 1,000 person-years for people aged ≥65 years.^10^ Fiest et al. conducted a meta-analysis and reported that the incidence of Alzheimer’s disease is 15.8 per 1,000 person-years among subjects aged ≥60 years.^11^ Based on these data, the incidence of α-synucleinopathies, such as Parkinson’s disease (8/57) and Lewy body dementia (3/57) in this study, during a mean follow-up period of 4 years, might be higher than that for the general population, although the incidence of Alzheimer’s disease in this study (n=2) might be comparable to that for the general population.

The meta-analysis performed by Galbiati et al. showed that the rate at which neurodegenerative disorders develop after a diagnosis of iRBD was extremely high, with 33.5% and 82.4% of patients developing disorders after 5 and 10.5 years, respectively.^3^ In the present study, the incidence of neurodegenerative disorders after a diagnosis of iRBD was slightly lower than that reported in the abovementioned meta-analysis, which may be due to this study being a retrospective study, in which the determination of neurodegenerative disorder onset was based on diagnoses from other departments during routine medical care, whereas several studies conducted overseas were designed as prospective studies, during which evaluations of cognitive function and neurological symptoms were systematically performed using uniform criteria. Therefore, the onset of neurodegenerative disorders may have been diagnosed later among the cohort in this study compared with those conducted overseas. Second, recently Japanese media, such as TV programs and newspapers, have frequently covered the transition from iRBD to neurodegenerative disorders, and the number of patients who seek medical care on their own accord for suspected RBD has been increasing. The time of iRBD diagnosis was used as the starting point for the observation period using the KM method in the present study, and a larger number of patients have been diagnosed with iRBD at a relatively early stage in Japan than in other countries; consequently, the incidence of neurodegenerative disorders may have been lower in this study than in others for similar after-diagnosis periods due to the early diagnosis of iRBD. The length of time between the onset of iRBD symptoms and the diagnosis of iRBD was not available among the present data; therefore, the contribution of early iRBD diagnosis to the differing results of these studies remains unclear. In addition, racial differences may contribute to the differences between studies. In the systematic review and meta-analysis performed by Galbiati et al., most of the included studies were conducted in Spain and Canada.^3^ For example, in a Canadian cohort study of 89 patients diagnosed with iRBD, Postuma et al. reported that 47% and 66% of patients developed neurodegenerative disorders 5 and 7.5 years after iRBD diagnosis, respectively.^12^ Iranzo et al. reported that 33.1% and 75.7% of patients with iRBD developed neurodegenerative disorders after 5 and 10 years, respectively, in a cohort study of 174 patients in Spain.^13^ In comparison, in the Asian region, Wing et al. reported that 8.5% and 38.1% of patients in China developed neurodegenerative diseases 5 and 9 years after RBD diagnosis, respectively, where the respective percentages were 9.2% and 52.6% among the 71 patients classified using strict criteria.^14^ Youn et al. reported that in Korea, 18% of 84 patients with iRBDs developed neurodegenerative disorders after 5 years.^15^ These discrepancies could be due to differences among the evaluation procedures; however, the frequency of developing neurodegenerative disorders after an iRBD diagnosis could also be slightly lower among Asian patients than among non-Asian patients.

Limited information is available regarding whether comorbid mental or sleep disorders affect the later incidence of neurodegenerative disorders in patients with iRBD. In a meta-analysis, Wang et al. reported that depression increases the risk of subsequent Parkinson’s disease diagnoses^16^; therefore, participants presenting with both iRBD and depression might have an increased risk for neurodegeneration. In this study, 5 of 57 patients had comorbid depression, and two of these (40%) later presented with neurodegenerative disorders. This ratio is numerically high; however, the survival curve (neurodegenerative disorder-free) for patients with depression was not statistically different from that for patients without depression (*P*=0.9118). In addition, sleep apnea is a risk factor for various diseases, and an association between sleep apnea and Parkinson’s disease has been suggested.^17^ In this study, 22 of 57 patients with iRBD had comorbid sleep apnea, of which five (22.7%) later presented with neurodegeneration. The survival curves for patients with and without sleep apnea did not significantly differ (*P*=0.7521). These results indicated that the high incidence of neurodegenerative disorders in this study may not be attributed to these comorbidities. Thus, further studies with larger sample sizes must be conducted to examine the additive risks caused by these comorbidities for the development of neurodegeneration in patients with iRBD.

This study has several limitations. The sample size and length of follow-up duration were modest compared with those reported by previous studies, which might affect the estimated incidence. The judgment of iRBD at the time of initial diagnosis did not depend on a systematic neurological evaluation; therefore, the possibility that latent neurodegenerative disorders were already present could not be completely excluded. If iRBD patients exhibited latent neurodegenerative disorders at the time of diagnoses, then the incidence later neurodegenerative disorder development might be even lower than estimated here. The diagnoses of neurodegenerative disorders were not based on uniform and standardized criteria, and the final diagnoses of neurodegenerative disorders were not confirmed via autopsy or histopathology, which may also affect the results. For example, patients with Alzheimer’s-type dementia might have also presented with the co-occurrence of Lewy body dementia. Because this study did not include a control group, the significance of a higher incidence of neurodegenerative disorders in patients with iRBD compared with those without could not be confirmed using the same methods.

Because the present study was retrospectively performed to determine the onset of neurodegenerative disorders and no standardized criteria were used for the evaluation of cognitive function and neurological symptoms, this study must be classified as preliminary. If a systematic follow-up were to be conducted, the incidence of neurodegenerative disease development in iRBD patients might be even higher. In the future, prospective studies using standardized evaluations and onset criteria must be performed in Japan. However, this study indicates that physicians who manage patients with sleep disorders and those who might initially diagnose patients with iRBD, should be aware of the high risk of neurodegeneration among this population, must be able to explain the risk adequately to patients, and must be willing to discuss follow-up procedures after iRBD diagnoses.

## Conclusions

We retrospectively analyzed the onset of neurodegenerative disorders in patients diagnosed with iRBD. The findings indicated the high likelihood of iRBD progressing to neurodegenerative disorders, even in Japan. This result is comparable with those reported by studies performed overseas. However, this study was a retrospective analysis, and standardized methods must be used to conduct prospective evaluations in Japan in the future.

## Figures and Tables

**Table1:** Patient Characteristics

	Total patients with initially diagnosed iRBD	Patients with later developed neurodegenerative disorders
Number of patients	57	14
Age at PSG, years (mean±SD)	65.6±7.45	59.2±17.1
Sex, men/women (%female)	47/10 (17.5%)	10/4 (28.6%)
Comorbid sleep disorders (%)	23 (40.3%)	6 (46.1%)
	SA 22, RLS 1, DA 1	SA 5, RLS 1
Comorbid mental disorders (%)	6 (10.5%)	2 (14.3%)
	MDD 5, panic disorder 1	MDD 2
Duration since iRBD diagnosis to last visit or onset of neurodegeneration, years (mean±SD)	4.0±2.8	5.9±2.0

PSG, polysomnography; SD, standard deviation; SA, sleep apnea; RLS, restless leg syndrome; DA, disorder of arousal; MDD, major depressive disorder; iRBD, idiopathic rapid eye movement sleep behavior disorder Note: One patient among total patients had two overlapped comorbid sleep disorders (sleep apnea and disorder of arousal).
